# Glucagon Test Is a Useful Predictor of Withdrawal From Insulin Therapy in Subjects With Type 2 Diabetes Mellitus

**DOI:** 10.3389/fendo.2022.871660

**Published:** 2022-04-28

**Authors:** Yuichiro Iwamoto, Tomohiko Kimura, Fuminori Tatsumi, Toshitomo Sugisaki, Masato Kubo, Erina Nakao, Kazunori Dan, Ryo Wamata, Hideyuki Iwamoto, Kaio Takahashi, Junpei Sanada, Yoshiro Fushimi, Yukino Katakura, Masashi Shimoda, Shuhei Nakanishi, Tomoatsu Mune, Kohei Kaku, Hideaki Kaneto

**Affiliations:** Department of Diabetes, Endocrinology and Metabolism, Kawasaki Medical School, Kurashiki, Japan

**Keywords:** glucagon test, type 2 diabetes, endogenous insulin secretion, beta-cell function, short-term insulin therapy, insulin withdrawal, HOMA-β, CPR

## Abstract

There are many tests for evaluating endogenous insulin secretory capacity. However, there are only a limited number of studies that have examined in detail in clinical practice which method most accurately reflects the ability to secrete endogenous insulin especially in hyperglycemic state. The purpose of this study was to find the endogenous insulin secretory capacity and a possible predictor of insulin withdrawal in subjects with type 2 diabetes requiring hospitalization due to hyperglycemia. In the endogenous insulin secretory test during hospitalization, CPR, CPR index, and ΔCPR after glucagon loading were all significantly higher in the insulin withdrawal group. On the other hand, there were no difference in fasting CPR index, HOMA-β, SUIT, and 24-hour urinary CPR excretion between the two groups. In the glucagon test of the insulin withdrawal group, the cutoff value of ΔCPR was 1.0 ng/mL, the withdrawal rate of ΔCPR of 1.0 ng/mL or more was 69.2%, and the withdrawal rate of less than 1.0 ng/mL was 25.0%. In conclusion, it is likely that glucagon test is the most powerful tool for predicting the possibility of insulin withdrawal as well as for evaluating endogenous insulin secretory capacity in subjects with type 2 diabetes requiring hospitalization due to hyperglycemia.

## Introduction

Type 2 diabetes is a metabolic disease that causes chronic hyperglycemia due to decreased insulin action caused by decreased endogenous insulin secretory capacity of pancreatic β-cells and decreased insulin sensitivity (insulin resistance). Type 2 diabetes is usually caused by genetic factors and acquired factors such as overeating and lack of exercise. Glycemic control has been shown to be effective in preventing the development of various diabetic complications (1–3). It is also said that at the onset of type 2 diabetes, the function of pancreatic β-cells is already decresed to less than half of the normal level (4), and β-cell mass and function gradually decrease after the onset of diabetes. Poor glecemic control facilitates the deterioration of β-cell function.

In type 2 diabetes, treatment is generally started with oral antidiabetic drugs such as metformin. However, in patients with marked hyperglycemia, short-term insulin therapy is often used to release from glucose toxicity. As the result, recovery of β-cell function leads to withdrawal from insulin therapy (5). On the other hand, patients with depletion of endogenous insulin secretion need to continue insulin therapy (6). In addition, an accurate assessment of endogenous insulin secretion is crucial in determining whether insulin withdrawal is possible in the future. The assessment of endogenous insulin secretory capacity includes HOMA-β (7), fasting serum C-peptide (CPR), 24-hour urinary CPR excretion (8), CPR index (9, 10), glucagon test (11), secretory unit of islets in transplantation index (SUIT) (12), arginine tests and so on. HOMA-β, which is considered to be useful when the fasting blood glucose level is less than 140 mg/dL, is not always useful because the proportion of patients with poor glycemic control is high as a characteristic of patients admitted to our department. In addition, serum insulin measurement is generally performed by whole insulin, but it is difficult to evaluate it during insulin treatment. Regarding 24-hour urinary CPR excretion, a false low value may be shown in cases of decreased renal function or bacterial urine (13). In many cases, accurate assessment of endogenous insulin secretory capacity is difficult in such situations. Glucagon and arginine tests are effective in assessing insulin secretory capacity accurately, and a ΔCPR of less than 1.0 ng/mL in the glucagon test indicates that endogenous insulin secretory capacity is decreased (9). Based on the above background, we performed glucagon test for patients whose insulin secretory capacity was difficult to evaluate by other methods.

To the best of our knowledge, few studies have investigated the relationship between ΔCPR in glucagon test and withdrawal from insulin therapy. Therefore, we conducted a retrospective study about the possible association between the results of the glucagon test and the withdrawal rate from insulin therapy 6 months after discharge.

## Materials and Methods

### Study Subjects

**Figure 1:**
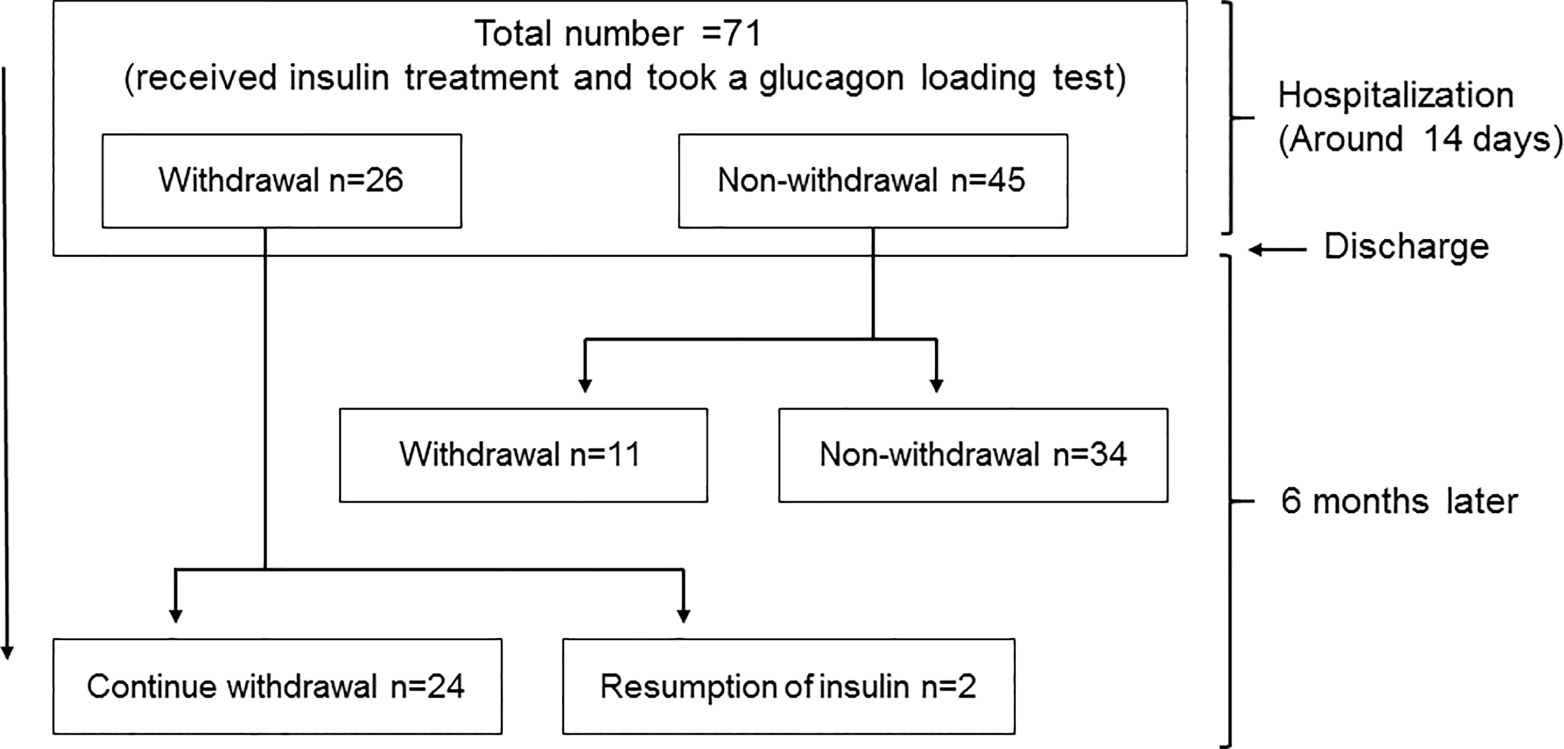
Study schema.

We performed this study with hospitalized patients retrospectively in our institution from April 1st in 2018 to March 31st in 2021. The study protocol including the Opt-out informed consent was approved by Institutional Review Board of Kawasaki Medical School (No.5295-00). And the study was conducted in accordance with the Declaration of Helsinki. Since this study was retrospective, instead of obtaining informed consent from each patient, we provided public information about the study *via* the hospital homepage. We initially selected 108 patients who required a glucagon test. Type 1 diabetes (2 patients), pancreatic diabetes (7 patients), unknown diabetes type (4 patients), using corticosteroids (2 patients), using immunosuppressive drugs (1 patient), insulinoma (4 patients), fasting blood glucose level 200 mg/dL or higher (9 patients), and diabetic nephropathy at stage 4 or higher (5 patients) were excluded. Finally, 71 patients with type 2 diabetes were included (**Figure 1**).

Insulin withdrawal rate was evaluated 6 months after discharge. We examined 35 patients who were able to withdraw insulin 6 months after discharge and 36 patients who were unable to withdraw (**Figure 1**).

### Method

On admission, diabetes- and lipid-related parameters, liver and renal function, blood pressure, body weight, grip strength, waist circumference, neck circumference, lower leg circumference were evaluated. BMI was calculated as weight (kg)/height squared(m^2^). During hospitalization, intensive insulin therapy was administered to all patients.

The glucagon test was performed in cases where a more detailed evaluation of endogenous insulin secretion was considered necessary, such as in cases where there was a discrepancy between clinical background and fasting CPR or 24-hour urinary CPR excretion. In the glucagon test, fasting blood glucose, serum CPR, and serum IRI were measured after fasting for more than 12 hours. Then, 1 mg of glucagon was administered intravenously, and the same measurements were taken 6 minutes later. CPR index was evaluated before and 6 minutes after glucagon loading. CPR index was calculated as serum CPR (ng/mL)/blood glucose (mg/dL) × 100. ΔCPR was calculated as serum CPR (ng/mL) after 6 minutes of glucagon loading – serum CPR (ng/mL) before glucagon loading.

To evaluate endogenous insulin secretory capacity other than the glucagon test, fasting blood glucose, fasting serum insulin, fasting serum CPR and 24-hour urinary CPR excretion were measured after adequate insulin therapy. Based on the results obtained, HOMA-β was calculated as (fasting insulin level (μIU/mL) × 360)/(fasting blood glucose level (mg/dL) - 63), and SUIT was calculated as 1500 × fasting serum CPR (ng/mL)/(fasting blood glucose level (mg/dL) - 61.7).

Patients who were withdrawn from insulin therapy 6 months after discharge were defined as a withdrawal group, and those who continued were defined as a non-withdrawal group. The criteria for withdrawal from insulin therapy were based on the judgment of the attending physician. The decision to withdraw from insulin therapy was based on insulin use of 0.2-0.3 units/kg/day and fasting blood glucose of less than 130 mg/dL and postprandial 2-hour blood glucose of 180 mg/dL. If the criteria were not met, weaning was still attempted base on the judgement of attending physician.

### Statistical Analysis

The clinical characteristics of the subjects used in the analysis were age, duration of diabetes, laboratory findings on admission, and insulin secretory capacity including glucagon test. Between-group differences in each measure between the withdrawal and non-withdrawal groups were analyzed using a Student’s t-test. ROC curves were generated for the contribution of each index of insulin secretory capacity to the withdrawal rate from insulin therapy during the first 6 months after discharge and were used for analysis. The contribution of medication use to insulin withdrawal was analyzed using logistic analysis. Statistical software was Excel Statistics for Mac version 16.54 (Social Research Information Co., Ltd., Tokyo, Japan) and JMP version 16.0.1 (SAS Institute Inc., Tokyo, Japan).

## Results

### Clinical Characteristics

The clinical characteristics of the patients are shown in **Table 1**. Diabetes-related parameters on admission were as follows: HbA1c (National Glycohemoglobin Standardization Program [NGSP]), 11.3 ± 2.7% and 11.0 ± 2.7%; glycoalbumin 32.3 ± 9.5% and 32.6 ± 2.7% in withdrawal and non-withdrawal group, respectively, suggesting the presence of marked hyperglycemia in both groups. There was no difference in HbA1c and glycoalbumin levels between the two groups. Duration of diabetes was significantly shorter and age was significantly younger in withdrawal group compared to that of non-withdrawal group. Neck and low leg circumferences were significantly longer in withdrawal group. In addition, withdrawal group tended to have a larger body mass index (BMI) than the non-withdrawal group. LDL-cholesterol was higher in withdrawal group compared to non-withdrawal group. Before hospitalization, 34% of the withdrawal group and 53% of the non-withdrawal group were using insulin preparation. The maximum daily insulin use during hospitalization was 31.5 ± 19.9 units/day (0.44 ± 0.23 units/kg/day) in the withdrawal group and 28.1 ± 11.8 units/day (0.46 ± 0.22 units/kg/day) in the non-withdrawal group, respectively. The mean time to insulin withdrawal in the withdrawal group was 32.4 days. There were 11 patients in the withdrawal group who required insulin therapy at discharge, and the number of units at discharge was 15.9 ± 11.5 units/day (0.18 units/kg/day). In addition, diabetic microangiopathies in this study subjects were as follows: neuropathy, 62.0%; retinopathy, 29.6%; nephropathy, 45.1%. Medication in this study subjects were: insulin, 43.7%; sulfonylurea or glinide, 26.8%; incretin-related drugs, 56.3%; biguanide, 29.6%, thiazolidine, 11.3%; alpha-glucosidase inhibitors, 9.9%; SGLT2 inhibitors, 19.7%.

**Table 1:** Comparison of various values between withdrawal group and non-withdrawal group on admission.

Parameter	All subjects (n = 71)	Withdrawal group (n = 35)	Non-withdrawal group (n = 36)	p value
Male/female	44/27	21/14	23/13	
Age (years)	64.3 ± 14.3	60.0 ± 16.2	68.6 ± 10.6	<0.05
Duration of diabetes (years)	16.3 ± 11.8	10.3 ± 9.4	22.2 ± 10.9	<0.005
Body weight (kg)	66.4 ± 16.6	70.0 ± 17.8	63.0 ± 14.6	n.s
BMI (kg/m^2^)	25.5 ± 6.4	25.9 ± 4.6	25.2 ± 7.8	n.s
Grip strength (kg)	24.1 ± 11.1	26.3 ± 12.4	21.7 ± 8.7	n.s
Waist circumference (cm)	91.1 ± 15.2	91.3 ± 13.0	90.9 ± 17.0	n.s
Neck circumference (cm)	36.8 ± 3.8	37.9 ± 3.8	35.6 ± 3.5	<0.05
Lower leg circumference (cm)	35.1 ± 4.0	36.2 ± 4.4	33.9 ± 3.2	<0.05
Blood glucose on admission (mg/dL)	181.0 ± 72.0	190.0 ± 89.7	172.2 ± 47.4	n.s
HbA1c (%, NGSP)	11.1 ± 2.7	11.3 ± 2.7	11.0 ± 2.7	n.s
Glycoalbumin (%)	32.5 ± 10.2	32.3 ± 9.5	32.6 ± 2.7	n.s
AST (U/L)	21.9 ± 7.8	21.8 ± 7.8	22.0 ± 7.8	n.s
ALT (U/L)	25.4 ± 14.3	26.2 ± 16.3	24.6 ± 11.9	n.s
Creatinine (mg/dL)	0.90 ± 0.45	0.85 ± 0.41	0.95 ± 0.48	n.s
BUN (mg/dL)	19.9 ± 11.3	19.1 ± 14.2	20.7 ± 7.4	n.s
Total cholesterol (mg/dL)	191.9 ± 50.0	205.0 ± 49.0	179.3 ± 47.5	<0.05
Triglyceride (mg/dL)	130.5 ± 76.4	144.9 ± 96.7	116.6 ± 45.1	n.s
LDL-cholesterol (mg/dL)	115.9 ± 41.7	128.1 ± 43.7	104.1 ± 36.0	<0.05
HDL-cholesterol (mg/dL)	47.8 ± 11.6	47.0 ± 11.7	48.6 ± 11.5	n.s

Data presented as mean ± standard deviation. BMI, Body mass index; LDL-cholesterol, Low-density lipoprotein cholesterol; HDL-cholesterol, High-density lipoprotein cholesterol. NS, Not Significant.

### Endogenous Insulin Secretory Capacity

Various parameters of endogenous insulin secretory capacity in withdrawal and non-withdrawal groups are shown in **Table 2**. ΔCPR, CPR after glucagon loading, and CPR index after glucagon loading were significantly higher in the withdrawal group than in the non-withdrawal group. ΔCPR was 1.03 ± 0.10 ng/mL in males and 1.43 ± 0.13 ng/mL in females, showing a trend toward higher values in females (p<0.05). In contrast, there was no difference in 24-hour urinary CPR, fasting CPR, fasting CPR index, or HOMA-β between the two groups. Taken together, there were significant differences in all parameters after glucagon loading between the two groups, whereas there was no difference in all parameters which was evaluated without any load.

**Table 2:** Various parameters of endogenous insulin secretory capacity in this study participants.

Parameter	All subjects (n = 71)	Withdrawal group (n = 35)	Non-withdrawal group (n = 36)	p value
Fasting blood glucose (mg/dL)	136.2 ± 35.7	134.1 ± 41.0	138.2 ± 29.5	n.s
Fasting C-peptide (ng/mL)	1.54 ± 0.80	1.63 ± 0.73	1.46 ± 0.87	n.s
Fasting C-peptide index	1.17 ± 0.61	1.27 ± 0.63	1.07 ± 0.58	n.s
Blood glucose after glucagon loading (mg/dL)	153.1 ± 35.5	150.0 ± 40.0	156.4 ± 30.2	n.s
C-peptide after glucagon loading (ng/mL)	2.73 ± 1.30	3.05 ± 1.14	2.41 ± 1.38	<0.05
C-peptide index after glucagon loading	1.83 ± 0.91	2.11 ± 0.90	1.55 ± 0.83	<0.05
ΔCPR (ng/mL)	1.19 ± 0.70	1.43 ± 0.67	0.95 ± 0.66	<0.05
HOMA-β (%)	22.9 ± 16.2	25.7 ± 15.6	19.9 ± 16.3	n.s
SUIT	35.8 ± 23.5	39.0 ± 22.6	33.2 ± 24.1	n.s
24-hour urine C-peptide (μg/day)	37.2 ± 23.1	36.8 ± 24.5	37.5 ± 21.7	n.s

Data presented as mean ± standard deviation. ΔCPR, delta C-peptide; HOMA-β, homeostatic model assessment beta cell function; SUIT, secretory unit of islet transplantation. NS, Not Significant.

### Predictors of Withdrawal From Insulin Therapy

**Figure 2:**
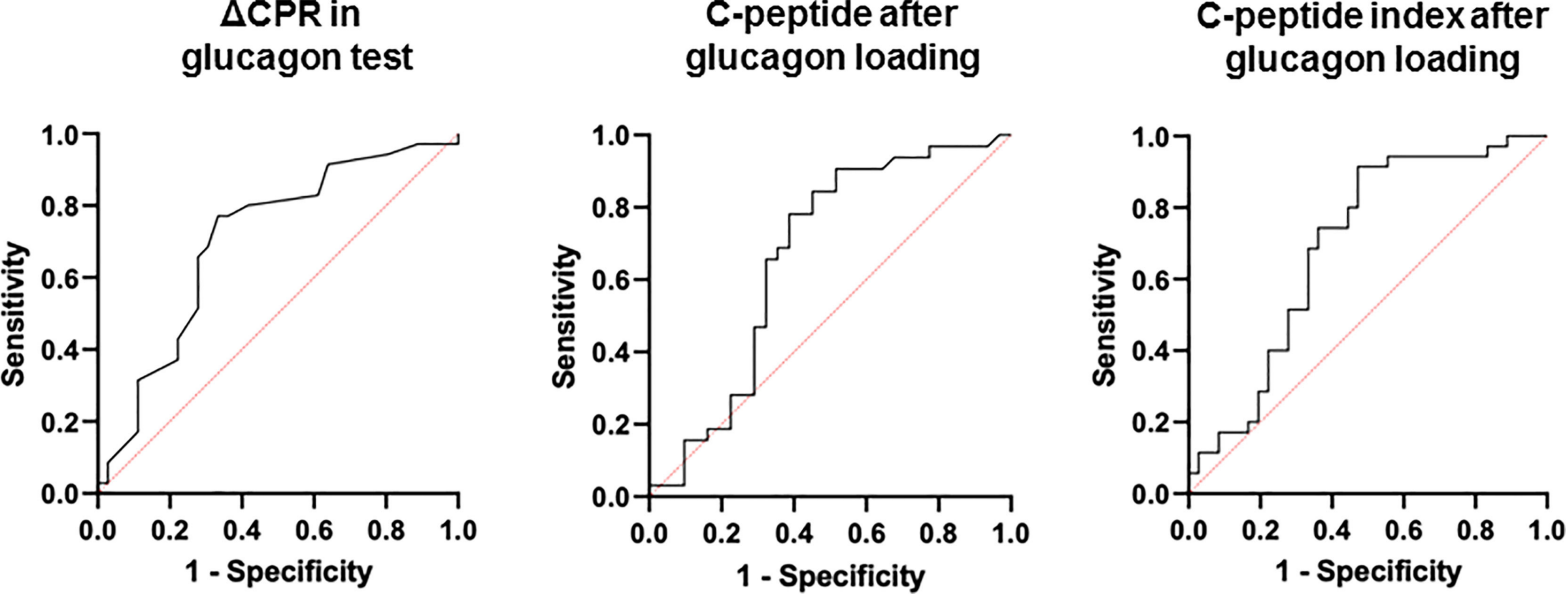
ROC curves to determine cutoff values of each indicator for withdrawal from insulin therapy.

ROC curves were constructed and analyzed to determine the cutoff values for each index of endogenous insulin secretory capacity with respect to withdrawal from insulin therapy (**Table 3** and **Figure 2**). Significant differences were observed in ΔCPR, CPR after glucagon loading, and CPR index after glucagon loading. The odds ratio for ΔCPR was 2.888 (95%CI 1.393 to 6560, P = 0.0068), the odds ratio for CPR after glucagon loading was 1.486 (95%CI 1.025 to 2.234, P = 0.044), and the odds ratio for CPR index after glucagon loading was 2.152 (95%CI 1.214 to 4.172, P = 0.014). The ROC curves were analyzed, and the cutoff values of the glucagon challenge test to predict insulin withdrawal in our institution were as follows: ΔCPR, 1.00 ng/mL (sensitivity 77.1%, specificity 66.7%); CPR after glucagon challenge, 2.30 ng/mL (sensitivity 77.1%, specificity 58.3%); CPR index after glucagon challenge, 1.25 ng/mL (sensitivity 91.4%, specificity 52.8%). Other parameters were insufficient to predict insulin withdrawal. Logistic regression analysis was also performed including age, gender, duration of diabetes, neck circumference, lower leg circumference and ΔCPR as explanatory variables. As a result, ΔCPR and duration of diabetes mellitus were considered independent factors in predicting withdrawal from insulin therapy (**Table 4**).

**Table 3:** ROC curves to determine cutoff values of each indicator for withdrawal from insulin therapy.

Parameter	AUC	p value	Cut-off	Sensitivity	Specificity	Odd ratios
Fasting blood glucose (mg/dL)	0.60675	n.s	141.0	80.0	55.6	0.997
Fasting C-peptide (ng/mL)	0.59167	n.s	1.00	85.7	36.1	1.299
Fasting C-peptide index	0.60873	n.s	0.56	100.0	25.0	1.753
Blood glucose after glucagon loading (mg/dL)	0.61746	n.s	155.0	77.1	58.3	0.996
C-peptide after glucagon loading (ng/mL)	0.66508	<0.05	2.30	77.1	58.3	1.486
C-peptide index after glucagon loading	0.69286	<0.05	1.25	91.4	52.8	2.152
ΔCPR (ng/mL)	0.70476	<0.05	1.00	77.1	66.7	2.888
HOMA-β (%)	0.64315	n.s	14.8	84.4	54.8	1.023
SUIT	0.63810	n.s	25.6	77.1	55.6	1.012
24-hour urine C-peptide (μg/day)	0.54167	n.s	35.6	65.7	58.3	0.999

ΔCPR, delta C-peptide; HOMA-β, homeostatic model assessment beta cell function; SUIT, secretory unit of islet transplantation. NS, Not Significant.

**Table 4:** Independent factors predicting withdrawal from insulin therapy in logistic regression analysis.

Parameter	Chi-square value	p value
Male/female	0.03	n.s
Age (years)	0.08	n.s
Duration of diabetes (years)	9.44	<0.005
BMI (kg/m^2^)	0.59	n.s
Neck circumference (cm)	1.21	n.s
Lower leg circumference (cm)	0.32	n.s
ΔCPR (ng/mL)	4.85	<0.05

BMI, Body mass index; ΔCPR, delta C-peptide. NS, Not Significant.

### Glucagon Test

Using the calculated cutoff values, we analyzed the insulin withdrawal rate after 6 months. First, we examined patients with a ΔCPR of 1.00 ng/mL or higher, and found that the withdrawal rate from insulin therapy was 69.2%. Patients who failed to withdraw from insulin despite high ΔCPR were characterized by older age and longer duration of diabetes (**Table 5**).

**Table 5:** Clinical characteristics of patients with ΔCPR of 1.0 ng/mL or higher.

Parameter	All subjects (n = 39)	Withdrawal group (n = 27)	Non-withdrawal group (n = 12)	p value
Male/female	19/20	14/13	5/7	
Age (years)	63.2 ± 15.3	60.2 ± 16.3	70.1 ± 9.6	n.s
Duration of diabetes (years)	14.3 ± 11.3	11.1 ± 10.0	21.5 ± 10.9	<0.05
Body weight (kg)	69.4 ± 19.4	69.3 ± 19.0	69.5 ± 20.1	n.s
BMI (kg/m^2^)	27.1 ± 7.4	25.7 ± 4.7	30.1 ± 10.6	n.s
Grip strength (kg)	22.2 ± 10.8	23.8 ± 11.3	17.7 ± 7.7	n.s
Waist circumference (cm)	94.1 ± 17.8	91.0 ± 14.0	101.0 ± 22.7	n.s
Neck circumference (cm)	37.4 ± 3.9	37.7 ± 3.9	36.6 ± 3.8	n.s
Lower leg circumference (cm)	35.5 ± 4.6	36.1 ± 4.8	34.2 ± 3.8	n.s
Blood glucose on admission (mg/dL)	189.9 ± 85.4	186.7 ± 95.4	197.3 ± 56.0	n.s
HbA1c (%, NGSP)	11.5 ± 2.7	11.2 ± 2.6	12.1 ± 3.0	n.s
Glycoalbumin (%)	32.9 ± 10.5	31.2 ± 9.2	37.2 ± 12.3	n.s
AST (U/L)	21.2 ± 7.5	22.1 ± 8.1	19.1 ± 5.2	n.s
ALT (U/L)	25.2 ± 14.7	26.9 ± 16.2	22.3 ± 9.4	n.s
Creatinine (mg/dL)	0.79 ± 0.36	0.83 ± 0.39	0.72 ± 0.28	n.s
BUN (mg/dL)	18.8 ± 12.3	18.4 ± 14.3	19.8 ± 5.6	n.s
Total cholesterol (mg/dL)	202.1 ± 47.9	200.6 ± 46.3	205.5 ± 51.0	n.s
Triglyceride (mg/dL)	140.3 ± 88.6	143.6 ± 100.6	132.8 ± 51.7	n.s
LDL-cholesterol (mg/dL)	123.8 ± 40.5	125.2 ± 42.6	120.8 ± 35.1	n.s
HDL-cholesterol (mg/dL)	48.7 ± 12.2	46.3 ± 11.8	53.9 ± 11.2	n.s

Data presented as mean ± standard deviation. BMI, Body mass index; LDL-cholesterol, Low-density lipoprotein cholesterol; HDL-cholesterol, High-density lipoprotein cholesterol. NS, Not Significant.

Next, we examined patients with ΔCPR less than 1.00 ng/mL and found that the withdrawal rate from insulin therapy was 25.0%. Patients who successfully withdrew despite low ΔCPR had a shorter duration of diabetes and significantly higher grip strength and BMI (**Table 6**).

**Table 6:** Clinical characteristics of patients with ΔCPR of less than 1.0 ng/mL.

Parameter	All subjects (n = 32)	Withdrawal group (n = 8)	Non-withdrawal group (n = 24)	p value
Male/female	24/8	7/1	17/7	
Age (years)	65.8 ± 12.9	59.3 ± 15.7	67.9 ± 11.0	n.s
Duration of diabetes (years)	18.9 ± 11.9	7.5 ± 6.6	22.6 ± 10.8	<0.005
Body weight (kg)	62.9 ± 11.5	72.3 ± 12.5	59.7 ± 9.2	<0.05
BMI (kg/m^2^)	23.6 ± 4.3	26.5 ± 4.1	22.7 ± 4.0	<0.005
Grip strength (kg)	26.5 ± 10.9	34.6 ± 12.2	23.4 ± 8.5	<0.05
Waist circumference (cm)	87.1 ± 9.6	92.8 ± 9.1	85.3 ± 9.0	n.s
Neck circumference (cm)	36.1 ± 3.6	38.6 ± 3.4	35.1 ± 3.2	<0.05
Lower leg circumference (cm)	34.6 ± 3.2	38.6 ± 3.4	35.1 ± 3.2	<0.05
Blood glucose on admission (mg/dL)	170.1 ± 49.0	201.3 ± 65.7	159.7 ± 36.5	<0.05
HbA1c (%, NGSP)	10.7 ± 2.6	11.7 ± 3.1	10.4 ± 2.3	n.s
Glycoalbumin (%)	31.9 ± 9.7	36.6 ± 9.3	30.4 ± 9.4	n.s
AST (U/L)	22.8 ± 8.1	20.9 ± 6.5	23.5 ± 8.4	n.s
ALT (U/L)	25.6 ± 13.9	23.8 ± 16.7	26.2 ± 12.7	n.s
Creatinine (mg/dL)	1.04 ± 0.50	0.96 ± 0.44	1.06 ± 0.51	n.s
BUN (mg/dL)	21.3 ± 9.8	21.6 ± 13.6	21.1 ± 8.2	n.s
Total cholesterol (mg/dL)	179.5 ± 49.6	219.8 ± 54.7	166.1 ± 39.6	<0.05
Triglyceride (mg/dL)	116.7 ± 56.0	149.1 ± 82.1	108.5 ± 39.0	n.s
LDL-cholesterol (mg/dL)	106.3 ± 41.2	137.8 ± 45.8	95.8 ± 33.5	<0.05
HDL-cholesterol (mg/dL)	46.8 ± 10.8	49.3 ± 10.9	45.9 ± 10.7	n.s

Data presented as mean ± standard deviation. BMI, Body mass index; LDL-cholesterol, Low-density lipoprotein cholesterol; HDL-cholesterol, High-density lipoprotein cholesterol. NS, Not Significant.

Withdrawal rates from insulin therapy for ΔCPR greater than or less than 1.0 were 69.2% and 25.0%, respectively (p<0.005, chi-square test). Patients who may be able to withdraw from insulin therapy with ΔCPR above or below 1.00 were characterized by younger age and shorter duration.

### Association of Drugs With Withdrawal From Insulin Therapy

The association between withdrawal from insulin therapy and medications used at the beginning and end of the study was analyzed using logistic regression analysis. The objective variable was the presence or absence of withdrawal from insulin therapy, and the explanatory variables were age, gender, HbA1c, ΔCPR, sulfonylurea and glinide, incretin-related drugs, biguanide, and SGLT2 inhibitor. Thiazolidinediones and alpha-glucosidase inhibitors were excluded due to the small number of cases in which they were used. As the results, the use of incretin-related drugs at the beginning and the use of biguanide at the end were associated with insulin withdrawal. It is noted, however, that even after consideration of possible influence of medication, ΔCPR was the most significantly correlated with withdrawal of insulin therapy at both the beginning and end of this study. These data strengthened the current findings that glucagon test is a useful predictor of withdrawal from insulin therapy in subjects with type 2 diabetes mellitus.

## Discussion

The glucagon test, first reported by Faber et al. in 1977, is a test to evaluate endogenous insulin secretory capacity by measuring blood CPR during glucagon loading (11). The 24-hour urinary CPR <20 μg/day (14), fasting CPR <0.48 ng/m (15), and ΔCPR <1.0 ng/mL in glucagon test (11) have been used as indices of insulin dependence. The present study suggests that the glucagon test is more useful than other tests for assessing endogenous insulin secretory capacity in type 2 diabetic patients requiring hospitalization. Furthermore, this study clearly shows that several data obtained in glucagon test are predictive indicators of subsequent withdrawal from insulin therapy.

It has been reported that exogenous insulin does not inhibit endogenous insulin secretion or cause negative feedback as long as there is no insulin excess to induce hypoglycemia (16). CPR responsiveness is not affected by glucagon loading during insulin therapy. However, there are few previous reports on the evaluation of endogenous insulin secretory capacity under conditions of high glucose toxicity. In the present study, in addition to the glucagon challenge test, HOMA-β, fasting CPR, CPR index, 24-hour urine CPR, and SUIT were also examined, but there was no correlation at all between such parameters without any load and discontinuation of insulin therapy. In the state of strong glucose toxicity, pancreatic β-cells are gradually exhausted and endogenous insulin secretion capacity is temporarily decreased, which may have prevented accurate evaluation in each test. In addition, CPR clearance decreases under hyperglycemic conditions, and fasting CPR may be relatively high and 24-hour urinary CPR relatively low (17), and such a mechanism may have made accurate assessment of endogenous insulin secretory capacity difficult.

When treating patients with marked hyperglycemia requiring hospitalization, fortified insulin therapy or continuous intravenous insulin therapy is often the treatment of choice. In particular, diabetic ketoacidosis, hyperglycemic hyperosmolarity syndrome, and soft drink ketosis can lead to fatal outcomes if left untreated, thus requiring immediate relief from glucose toxicity in such conditions. Evaluation of endogenous insulin secretory capacity is essential for treatment selection after acute treatment. However, one of the concerns in the acute phase examination is that the metabolic environment is presumably very different from that of normal outpatient care, such as exhaustion of pancreatic β-cells due to glucose toxicity and administration of a large amount of exogenous insulin. In the present study, the glucagon test was performed 7 to 14 days after the start of treatment for marked hyperglycemia, and it is possible that tests other than the stress test did not show significant differences. In addition to the glucagon test, other methods to assess endogenous insulin secretory capacity include the oral glucose tolerance test (OGTT), the mixed meal tolerance test, and the arginine test. While these load tests have the potential to more accurately assess endogenous insulin secretion capacity, but they are not suitable for populations in this study with an average HbA1c of approximately 11%. We also believe that these tests were not appropriate at least in this study subjects due to ethical issues as well. In addition, OGTT is performed in principle for diagnosis of diabetes mellitus in subjects without confirmed diagnosis of the disease and this test is not performed in principle in subjects with confirmed diagnosis with diabetes mellitus at least in Japan.

There is a limitation in this study. Although we think that the data obtained in this study include useful information especially from the clinical point of view, the number of subjects in this study was quite small. Therefore, similar study with a larger population would be necessary to reconfirm the findings in this study, which we think would lead to strengthening our working hypothesis.

Taken together, although there have been studies evaluating glucagon test and endogenous insulin secretory capacity, to the best of our knowledge, this is the first report to clearly show the relationship between glucagon test and insulin withdrawal in subjects with type 2 diabetes mellitus. We should bear in mind that glucagon test is a very useful predictor of withdrawal from insulin therapy. In addition, since glucagon test is a very simple loading test, we should willingly perform glucagon test when needed for diabetes care in clinical practice.

## Data Availability Statement

The original contributions presented in the study are included in the article/supplementary material. Further inquiries can be directed to the corresponding author.

## Ethics Statement

The studies involving human participants were reviewed and approved by Institutional Review Board of Kawasaki Medical School. Written informed consent for participation was not required for this study in accordance with the national legislation and the institutional requirements.

## Author Contributions

YI and FT designed the study. YI, FT, TS, MK, EN, KD, RW, HI, KT, JS, YF and YK treated patients and collected data. YI analyzed the data. YI, TK, FT, TS, MS, SN, TM, KK, and HK contributed to discussion. KK supervised the project. YI and TK wrote the manuscript. HK reviewed and edited the manuscript. All the authors have reviewed the manuscript. All authors contributed to the article and approved the submitted version.

## Conflict of Interest

The authors declare that the research was conducted in the absence of any commercial or financial relationships that could be construed as a potential conflict of interest.

## Publisher’s Note

All claims expressed in this article are solely those of the authors and do not necessarily represent those of their affiliated organizations, or those of the publisher, the editors and the reviewers. Any product that may be evaluated in this article, or claim that may be made by its manufacturer, is not guaranteed or endorsed by the publisher.
